# Self-perception and knowledge of evidence based medicine by physicians

**DOI:** 10.1186/s12909-016-0681-6

**Published:** 2016-06-29

**Authors:** Karen A. Aguirre-Raya, María F. Castilla-Peón, Leticia A. Barajas-Nava, Violeta Torres-Rodríguez, Onofre Muñoz-Hernández, Juan Garduño-Espinosa

**Affiliations:** Universidad Nacional Autónoma de México (UNAM), México City, Mexico; Department of Medical Publications, Hospital Infantil de México Federico Gómez (HIMFG), National Health Institute, México City, Mexico; Evidence-Based Medicine Research Unit, Hospital Infantil de México Federico Gómez (HIMFG), National Health Institute, México City, Mexico; Direction of Research, Hospital Infantil de México Federico Gómez (HIMFG), National Health Institute, México City, Mexico; Direction of Research, Hospital Infantil de México Federico Gómez (HIMFG), Torre de Hemato-Oncología e Investigación, National Health Institute, Dr. Márquez, No. 162, Col. Doctores, Del. Cuauhtémoc, C.P. 06720 México, DF Mexico

**Keywords:** Evidence-based medicine, Self-perception, Knowledge

## Abstract

**Background:**

The influence, legitimacy and application of Evidence Based Medicine (EBM) in the world is growing as a tool that integrates, the best available evidence to decision making in patient care. Our goal was to identify the relationship between self-perception about the relevance of Evidence Based Medicine (EBM) and the degree of basic knowledge of this discipline in a group of physicians.

**Methods:**

A survey was carried out in a third level public hospital in Mexico City. Self-perception was measured by means of a structured scale, and the degree of knowledge through parameter or “rubrics” methodology.

**Results:**

A total of 320 questionnaires were given to 55 medical students (17 %); 45 pre-graduate medical interns (14 %); 118 medical residents (37 %) and 102 appointed physicians of different specialties (32 %).

Self-perception of EBM: The majority of those surveyed (*n* = 274, 86 %) declared that they were very or moderately familiar with EBM. The great majority (*n* = 270, 84 %) believe that EBM is very important in clinical practice and 197 physicians (61 %) said that they implement it always or usually. The global index of self-perception was 75 %.

*Knowledge of EBM*: *Definition of EBM*; Seven of those surveyed (2 %) included 3 of the 4 characteristics of the definition, 82 (26 %) mentioned only two characteristics of the definition, 152 (48 %) mentioned only one characteristic and 79 (25 %) did not include any characteristic of EBM. *Phases of the EBM process*: The majority of those surveyed (*n* = 218, 68 %) did not include the steps that characterize the practice of EBM, of which 79 participants (25 %) mentioned elements not related to it. The global index of knowledge was 19 %.

**Conclusions:**

The majority of the surveyed physicians have a high self-perception of the relevance of EBM. In spite of this, the majority of them did not know the characteristics that define the EBM and phases of the process for its practice. A major discrepancy was found between self-perception and the level of basic knowledge of EBM among the surveyed physicians.

**Electronic supplementary material:**

The online version of this article (doi:10.1186/s12909-016-0681-6) contains supplementary material, which is available to authorized users.

## Background

The concept of Evidence Based Medicine (EBM) was introduced in 1992 by Gordon Guyatt at McMaster University [1]. Its inclusion in medical practice is part of a universal cultural movement made up of an extension of scientific tradition into different areas of human activity. In 1996 David Sackett described it as the conscientious, explicit, and judicious use of current best evidence in making decisions about the care of individual patients. Sackett stipulates that EBM is the integration of three important aspects: the best available evidence, clinical experience and patient’s values [2–4].

The practice of EBM requires, according to its founders, the application of the following methodology: 1) identify a question of clinical interest, the reply to which can benefit a particular patient; 2) seek information to locate and obtain, as efficiently as possible, the best scientific evidence with which to respond to the question; 3) critically evaluate the validity of said evidence (proximity to the truth), impact (strength of the effect) and applicability (usefulness in clinical practice); 4) integrate the critical evaluation with the biological data of the patient, his/her values and particular circumstances; 5) evaluate the fulfillment of the previous steps and find ways to improve the process next time [5].

Perception has been defined as the personal view that an individual has of him/herself and of reality. It is construed based on cognitive processes and on the personal sense of person’s experience [6]. Self-perception more specifically refers to an ample and coherent pattern of beliefs related to the manner in which one perceives oneself [7]. Since EBM seeks to incorporate scientific thinking to patient care, the self-perception of physicians about their knowledge of this discipline is of fundamental importance for an effective learning experience, one which can be generalized and even transferred to a different context [8].

Floyd Allport stated that perception is something that includes the acknowledgment of the elements of the environment in all its complexity, as well as that of each of the objects that constitute it. On the other hand, has been mentioned, that acknowledgment of objects is more attributable to the cognition than perception, however, both processes are so closely interrelated that, from the theoretical point of view, cannot be considered in isolation [9].

The objective of this study was to identify self-perception regarding the degree of familiarity with importance and applicability of EBM in a group of medical students, physicians in different stages of their training and medical specialists with an ongoing hospital clinical practice, in order to establish their relationship with their degree of knowledge of the concept and general method of EBM.

## Methods

### Design

#### Analytical cross-sectional study

The study took place at a third level of care public hospital in Mexico City. This hospital gives highly complex health services with medical and surgical specialties among others, targeted to solving problems of people sent by First and Second level establishments, or those who come spontaneously out of emergency. A survey was given to medical students, physicians in different stages of their training and medical specialists with an ongoing hospital clinical practice. The sampling was done for convenience, while maintaining the proportions of staff interviewed based on their employment or academic status. Information was collected on: sex, age, preceding university and work status (physician with hospital appointment, resident, pre-graduate intern, medical student). The field of specialization was recorded for physicians with a hospital appointment and residents. This group was labeled “graduate physicians”. All of the pre-graduate interns carried out clinical activity as part of their training and the medical students were undergoing their clinical practice cycle. This ensured that they had daily contact with patients, and this group was labeled “physicians in training”.

### Survey

A survey drafted in Spanish, comprised by three sections. On the first section, the data included allowed interviewee characterization. The second and third sections were focused on exploring the elements suggested in this research and consisted of five questions. The second section contained three closed questions regarding self-perception, using a Likert scale [10]: a) degree of familiarity with EBM (high, medium, some, none); b) how important does the subject feel that EBM is for his/her clinical practice (very important, somewhat important, not important); c) frequency with which the subjects apply EBM (always, usually, sometimes, never). The third section consisted of two open questions to explore true knowledge of fundamental and basic aspects of EBM, including its definition and the phases it is comprised with. All questions regarding knowledge were prepared based on the outlook of the group that gave rise to this discipline (Sackett et al.), taking into account the definitions expressed in different forums and documents by this group, including the original publication in 1992 [1–3, 5].

#### Validation of the survey and pre-test

The survey was discussed and validated for its appearance and content by a group of three researchers well known for their experience in the field, all of them university professors in the area of Evidence Based Medicine at the pre-graduate and post-graduate level. In addition, a pre-test was done in order to establish that the potential study population understood the questions. Those questions that did not provide relevant information for the study, as well as those that were potentially confusing to the subjects were excluded.

#### Rubrics

In order to encode and evaluate open ended questions that explored knowledge of EBM in terms of its definition and phases, parameters or “rubrics” were established [11]. These “rubrics” constitute an analytical strategy that allows for the taking apart of a concept in order to identify different elements related to the subject at hand. The rubrics are thus guides that evaluate what has been learned about specific subjects; for example, for the question about the definition of EBM four rubrics were used, one for each of the elements that make up Sackett’s original definition (1996) [2, 3]. One point was assigned for each rubric when the evaluated element was contained in the subject’s answer or a similar idea was presented. In this manner, the total score could be anywhere from zero to four points. Similar weighting to the rubrics included is based on insufficient empirical information and insufficient theoretical development, that would assign different values to the elements on which the discipline is based.

The same technique was applied to the question regarding the phases of EBM, which contained six rubrics. Each rubric was related to each one of the phases of the process, as they were originally described by Sackett et al. [5]. Except for the fourth phase which includes two very important aspects, and two rubrics were assigned to it because of this. The weighing of this was similar to that of the definition, for a total score of six points. Evaluation of the answers was done independently by two evaluators, with a kappa concordance index of between 0.74 and 0.97 for the different rubrics that were analyzed.

#### Self-perception index

In order to create an index that would permit observing the level of relevance that each physician gives to EBM, a points system was assigned to each of the response categories in the three questions intended for the analysis of self-perception by means of the Likert scale. Therefore, when greater relevance was declared for EBM the score was higher. The highest score for self-perception was 8 points, which indicates being very familiar with EBM, considering it to be very important, and always applying it in medical practice. This variable was categorized in four strata: high self-perception when the physician met between six and eight points; moderate, when met a score between three and five points; slight self-perception when obtained a score of one or two points. Self-perception regarding the relevance of EBM was considered null when the response was that there was no familiarity with EBM, that the discipline is unimportant, and that it was never applied in daily practice. A global self-perception index was obtained by dividing the total sum of the scores obtained for the three questions into the expected total points for each of the study groups.

#### Knowledge index

The sum of the questions regarding the definition and phases of EBM was a total of ten rubrics. The variable was categorized in four strata: high knowledge, those that obtained between seven to ten rubrics; moderate knowledge, between four and six rubrics; slight knowledge, between one and three rubrics and null knowledge when zero rubrics were had. To obtain the global knowledge index, the correct responses to the rubrics were added and then divided into the maximum expected total score in each group.

### Statistical analysis

The evaluation of concordance between the two evaluators of the rubrics was done by means of a kappa concordance index. Relationship between self-perception and knowledge was determined by the Spearman correlation coefficient (r_s_). Self-perception and knowledge of EBM were compared between the group of graduated physicians and the group of physicians in training, and the association between these two variables was determined by the odds ratio (OR) with a 95 % confidence interval. Statistical significance was calculated by the chi-square method. In order to test the hypothesis about differences in knowledge and self-perception among the groups of physicians the Mann Whitney *U* test was performed. The level of statistical significance was set at 0.05 (two-tailed).

## Results

Initially 360 physicians were planned to be surveyed, but 40 refused to participate (11 %). A total of 320 surveys were taken, of which 55 (17 %) were taken by medical students, 45 (14 %) were taken by pre-graduate interns, 118 (37 %) were taken by specialist residents and 102 (32 %) by physicians of different specialties.

A slight predominance of males was observed in the sample population (*n* = 180, 56 %), and the average age for the entire group was 34 years (SD = 14.5). The majority of those surveyed were graduates of or students at the Universidad Nacional Autónoma de México (UNAM) (*n* = 174, 54 %), other public universities were 103 that corresponds to 20 %, a smaller number came from private universities (*n* = 27, 8 %) and 16 participants (5 %) had studied in foreign universities (mainly in South American countries). The graduate physicians were appointed physicians and residents (*n* = 220, 69 %); given their great diversity, specialties were divided into medical (*n* = 124, 39 %), surgical (*n* = 62, 19 %) and other (*n* = 34, 11 %).

### Self-perception of EBM

The majority of those surveyed stated that they are very or quite familiar with EBM (*n* = 274, 86 %); graduate physicians mentioned a greater degree of familiarity than the physicians in training (OR = 2.77, 95 % CI = 1.68–4.5, *p* <0.001). They also gave a greater degree of importance to EBM than those physicians with lesser experience (OR = 1.93, 95 % CI = 1.04–3.5, *p* = 0.03). With regard to the use of EBM in daily clinical practice, 197 of the total physicians (61 %) stated that they always or generally use it (70 % of these were graduate physicians and 44 % were physicians in training). Among the total of physicians, 18 responded that they never use it (6 %) (Table 1).

**Table 1:** Self-perception of physicians regarding degree of familiarity, importance and applicability of Evidence Based Medicine (EBM)

Self-perception	Graduate physicians *n* = 220		Physicians in training *n* = 100		Total physicians *n* = 320	
	f	%	f	%	f	%
Degree of familiarity with EBM						
High	122	55	31	31	153	48
Medium	76	35	45	45	121	38
Some	19	9	21	21	40	12
None	3	1	3	3	6	2
Importance of EBM						
Very important	192	87	78	78	270	84
Somewhat important	25	11	22	22	47	15
Not important	3	1	0	0	3	1
Applies EBM in usual medical practice						
Always	66	30	18	18	84	26
Usually	87	40	26	26	113	35
Sometimes	58	26	47	47	105	33
Never	9	4	9	9	18	6

### Knowledge of the definition and phases of the process for using EBM

**Fig. 1:**
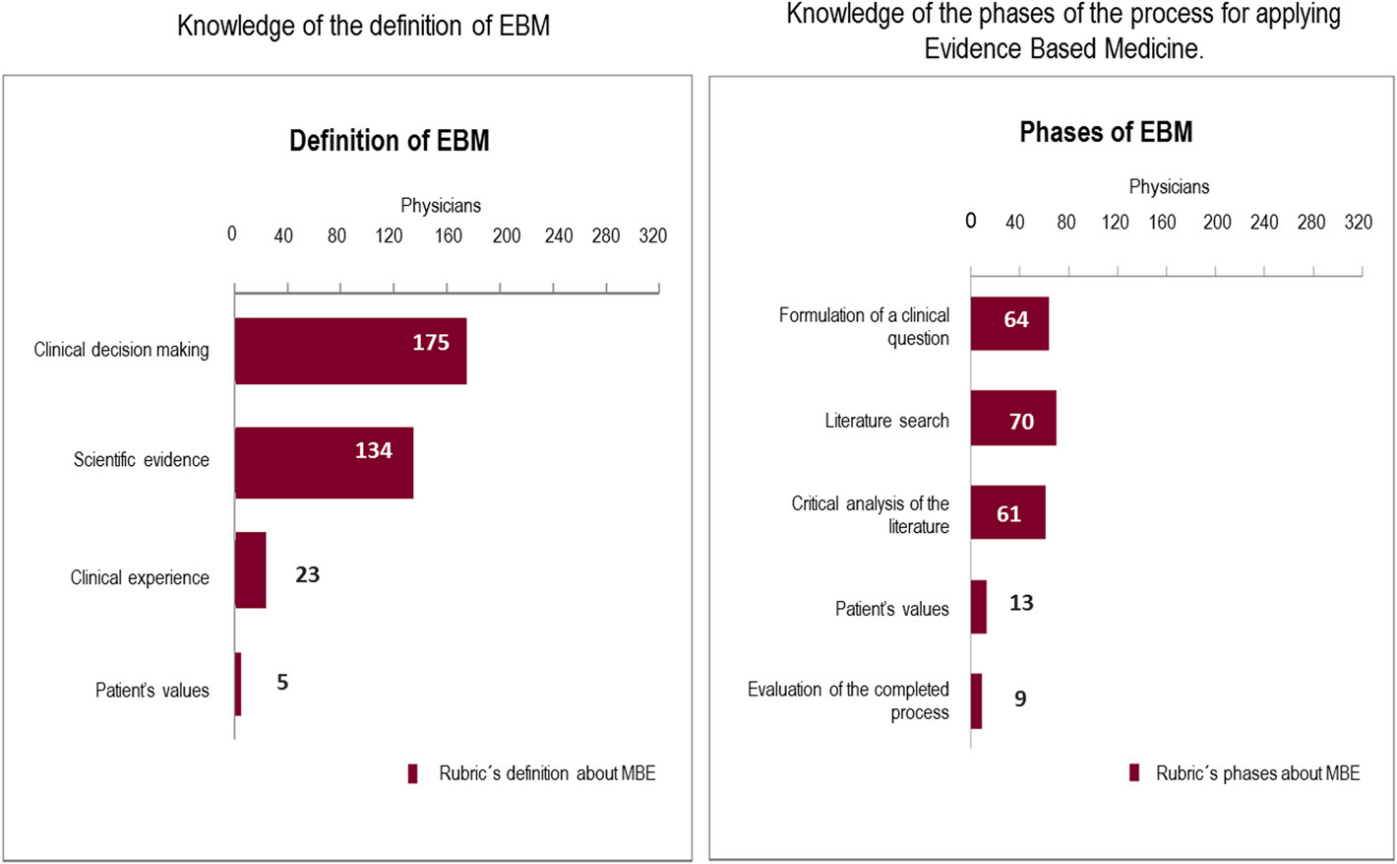
Knowledge of the definition and phases of the process for using Evidence-Based Medicine
- Phases of the EBM process. The majority of those interviewed (*n* = 218, 68 %) did not include any of the steps that characterize the use of EBM. Of these, 79 (25 %) mentioned elements that were not pertinent to the subject (Table 3). Five physicians were able to identify the six rubrics (the maximum number of rubrics that were established) that describe the EBM process. Between 3 and 5 rubrics were mentioned by 45 physicians (14 %), and one or two rubrics were identified by 52 of them (16 %).

- Definition of EBM. None of the participating physicians were able to identify the four distinctive characteristics of the conventional definition of EBM. Only 7 (2 %) of them included 3 of the 4 characteristics of the definition, 83 (26 %) mentioned 2 characteristics, and 150 (47 %) had one correct response. The rubric that referred to clinical decision making and/or use was most frequently mentioned (*n* = 175, 55 %), but instead the one that refers to the need to take into consideration the values and preferences of patients was only included by 5 physicians (2 %) (Table 2) (Fig. 1).

**Table 2:** Knowledge of the definition of Evidence Based Medicine

Evaluation parameters (rubrics)	Graduate physicians *n* = 220		Physicians in training *n* = 100		Total physicians *n* = 320	
	f	%	f	%	f	%
Clinical decision making and/or application	120	55	55	55	175	55^a^
Scientific evidence and/or Results	102	46	32	32	134	42
Clinical experience	17	78	6	6	23	7
Patient’s particular values and/or circumstances	3	1	2	2	5	2
Non-pertinent responses	50	23	25	25	74	23
No response	3	1	2	2	5	2

^a^The sum of percentages is not 100 % because the participants responded to more than one rubric

**Table 3:** Knowledge of the phases of the process for applying Evidence Based Medicine

Evaluation parameters (rubrics)	Graduate physicians *n* = 220		Physicians in training *n* = 100		Total physicians *n* = 320	
	f	%	f	%	f	%
Formulation of a clinical question	35	16	29	29	64	20^a^
Literature search	36	16	34	34	70	22
Critical analysis of the literature	31	14	10	10	61	19
Application of the information in clinical practice	24	11	28	28	52	16
Patient’s values and preferences	0	0	13	13	13	4
Evaluation of the completed process	2	1	7	7	9	3
Non-pertinent responses	59	27	19	19	79	25
No response	103	47	36	36	139	43

^a^The sum of the percentages is not 100 % because the participants responded to more than one heading

### Global index of self-perception and knowledge

#### Self-perception

**Fig. 2:**
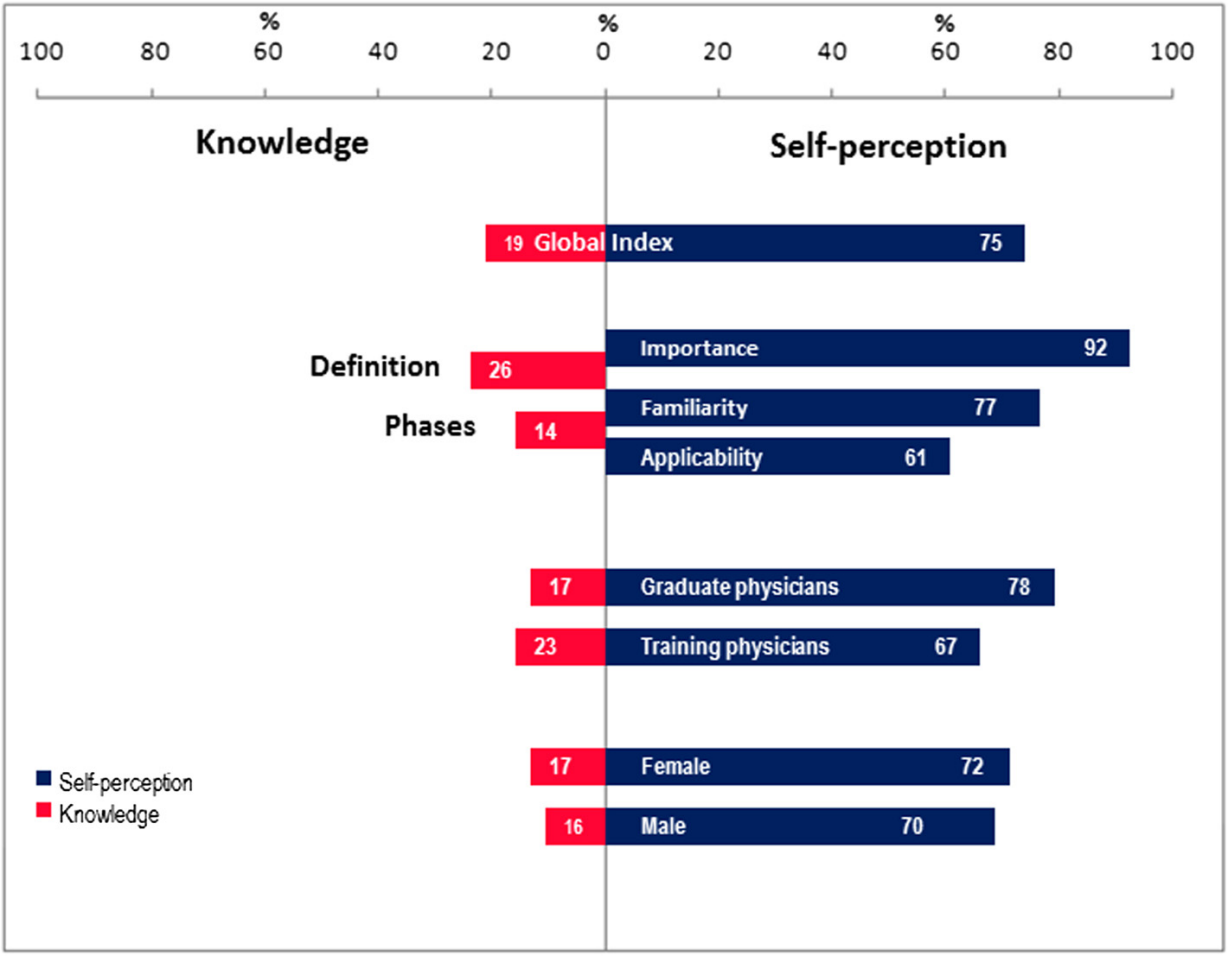
Global index of self-perception and knowledge

The global index for self-perception was 75 %, that is, 1911 points obtained out of the 2560 possible ones. When the items of familiarity, importance and use were considered jointly, 59 (18 %) participants had the highest global self-perception index (are very familiar with EBM, consider it very important and always use it). According to the established classification, 200 of the participants had a high self-perception (63 %). There was a greater degree of self-perception observed among graduated physicians than among those still in training (Table 4) (Fig. 2).

**Table 4:** Global index of self-perception about EBM

Index of self-perception		Graduate physicians *n* = 220		Physicians in training *n* = 100		Total physicians *n* = 320	
		f	%	f	%	f	%
High	8	48	22	11	11	59	18
	7	69	31	13	13	82	26
	6	40	18	19	19	59	18
Moderate	5	36	16	24	24	60	19
	4	15	7	23	23	38	12
	3	9	4	10	10	19	6
Slight	2	1	0.4	0	0	1	0.3
	1	0	0	0	0	0	0
Null	0	2	1	0	0	2	0.6

#### Knowledge

The global index for knowledge was 19 %, that is, only 602 rubrics out of the 3200 possible ones were obtained. None of the physicians provided all of the elements contained in the definition and process for the practice of EBM. The highest number of rubrics was 8 and was obtained by two of the participants (0.6 %). When the number of physicians who obtained four or more rubrics was considered, there were differences observed between graduated physicians (30/220, 14 %) and physicians in training (27/100, 27 %). Of the total of physicians, 62 % (198/320) only identified between one and three rubrics, and among those who did not identify any (*n* = 65, 20 %) there were different responses that were not considered pertinent to the identification of EBM (Table 5).

**Table 5:** Global index of knowledge of EBM

Knowledge Index		Graduate physicians *n* = 220		Physicians in training *n* = 100		Total physicians *n* = 320	
		f	%	f	%	f	%
Null	0	49	22	16	16	65	20
Slight	1	77	35	28	28	105	33
	2	47	21	23	23	70	22
	3	17	8	6	6	23	7
Moderate	4	10	5	10	10	20	6
	5	15	7	9	9	24	8
	6	1	0.4	4	4	5	2
High	7	3	1	3	3	6	2
	8	1	0.4	1	1	2	0.6

#### Hypothesis testing

The correlation between self-perception and knowledge was minimal (r_s_ = 0.02, 95 % CI = - 0.08–0.12, *p* = 0.62); on the other hand, was discreet but statistically significant in the group of medical graduates (r_s_ = 0.13, 95 % CI = 0.01–0.27, *p* = 0.04), not so in the group of physicians in training, (r_s_ = - 0.12, 95 % CI = - 0.36–0.06, *p* = 0.20) in which the correlation was negative. To establish the degree of association between the level of experience (graduated physicians vs. physicians in training) and self-perception, an association was estimated between having a high perception level (greater than 75 %) and being a graduated physician (OR = 3.59, 95 % CI = 2.11–6.11, *p* < 0.001). The hypothesis that being a graduated physician would be a risk factor for having scarce knowledge of the subject was tested; this hypothesis is based on the fact that physicians in training more frequently receive courses on the subject (A score of less than 50 % of the analyzed rubrics was considered to be a low level of knowledge) (OR = 3.73, 95 % CI = 1.19–11.7, *p* = 0.01). Statistically significant differences were found between the distribution of the self-perception scores in the two groups under study (graduated physician vs physicians in training) (95 % CI = -1.2–0.53, *p* < 0.001), as well as the distribution of the scores on knowledge (95 % CI = 0.15–0.95, *p* < 0.01).

## Discussion

The history of Science, with its content of values, suppositions, theory and methods has impacted medical practice through the birth of Clinical Epidemiology in the 1960′s, in the twentieth century. However, the adoption of scientific thinking in activities related to patient care has been slow; medical practice has remained mainly a heuristic activity, that is, it depends on experience and the art of problem solving.

The importance of Science in our society is accepted in a generalized manner, but learning it is a laborious process, which is why mastery of it is not often achieved [12]. For this reason the high self-perception of physicians regarding EBM (75 %) can be explained, in spite of the contrast with their scarce knowledge of the basics of the discipline (19 %). A high percentage of the participants in this study stated that they consider EBM to be very important, are very familiar with it and use it on a daily basis. In actuality, their knowledge and understanding of the principles on which it is based are quite scarce. The risk focus that was utilized provided evidence that being a graduated physician is associated with greater levels of self-perception, as well as with lower levels of knowledge.

A study of primary care physicians showed that 60 % of them were familiar with the concept of EBM, and 56 % stated that they had used it. When they were asked to name the components of EBM that differ from clinical experience, only 39 % of them were able to mention them [13]. Other studies have found that only a small proportion of physicians understand and are able to explain, the common epidemiological concepts that are used in EBM [14–16].

The existence of a positive illusory bias has been proposed to describe this human tendency to overestimate our capabilities. This bias consists of a disparity between the self-reporting of the competence that the subject believes he or she has and the competence that he/she actually has. In this way, the perception of one’s own capabilities is substantially greater than those which one actually has [17, 18]. According to Kahneman and Tversky, human beings tend to overestimate the exactitude of their opinions and judgements, so it is therefore easy to imagine that one is always right. Generally, in accordance with these authors, one tends to seek information that supports and reinforces what we believe, and we avoid that which contradicts us [19, 20].

It has been described that self-assessment of one’s knowledge is fundamental to reach efficient knowledge. For that, it is deemed crucial that the subject develops a self-perception of their own knowledge, one that reasonably fits reality, in order to reach effective knowledge which, on the other hand, could be generalized to different contexts [8].

The distribution according to the University of origin, reasonably reflects the participation of various universities in public hospitals in Mexico. In addition, the curricular content related to Medicine Based on Evidence in public and private universities, show some degree of variation and heterogeneity, however, generally they tend to incorporate aspects of a medical practice based on scientific evidence. In general, is worth noting that curricular content of participant Universities incorporates elements related with the Clinical Epidemiology or Evidence Based Medicine. Even though curricular contents have suffered variations over the past two decades, overall, they are now more uniform and stress scientific elements applied to medical practice.

This study is based on the premise that it is difficult to believe that someone can practice EBM without knowing its basic elements and the formal process that characterizes it. However, the study is limited by the fact that the analysis of the knowledge of this discipline among the participants was centered on only a few questions. One limitation of this study was not being able to perform random sampling to select participants. However, when conducting interviews, physicians were randomly selected among the ones who were working during the days the study was taking place. When selecting physicians tried to keep the proportion of medical graduates and training in relation to the actual proportion of such staff in the hospital analyzed. Although we believe that perception and the degree of knowledge is homogeneously distributed, it is a study limitation not count with a random sampling due to feasibility reasons.

On the other hand, the measuring instrument of perception and knowledge in this area requires a validation process more comprehensive and extensive. In this study, knowledge was restricted to explore issues related to the fundamentals of EBM; however, it would need to investigate further the knowledge related to the methodological bases and statistics of discipline, which in turn would allow a medical practice based on evidence. In addition, the survey sample is based in a hospital, which restricts the study’s external validity.

Our results support the hypothesis that health personnel overestimate their understanding of Evidence Based Medicine. In order to confirm this in a reasonable manner it would be necessary to enlarge the study groups and delve deeper into the levels of technical knowledge related to the practice of clinical science. It is suggested here that the required historical transition is not yet complete in the practice of clinical medicine, a transition that would allow science to be a part of different aspects of culture and human activity; because of this there is still a scarce understanding of scientific values and methods. This situation can be explained by the strength of present traditions, as well as by the delay in incorporating scientific thought, which is attributable to historical reasons and in the very nature of the discipline.

## Conclusions

The majority of the surveyed physicians have a high self-perception of the relevance of EBM. In spite of this, the majority of them did not know the characteristics that define the EBM and phases of the process for its practice. A major discrepancy was found between self-perception and the level of basic knowledge of EBM among the surveyed physicians.

## Abbreviations

EBM, evidence based medicine; OR, odds ratio; UNAM, Universidad Nacional Autónoma de México

## Additional files

Additional file 1: Survey. (PDF 22 kb) Additional file 2: Spreadsheet Self-perception and knowledge of EBM. (XLSX 87 kb)
