# Supplementary material for: Self-perception and knowledge of evidence based medicine by physicians
Source: BMC Med Educ. 2016 Jun 29;16:166. doi: 10.1186/s12909-016-0681-6 (PMC4928273; doi:10.1186/s12909-016-0681-6)
Supplement: Additional file 1: — Survey. (PDF 22 kb) [file 12909_2016_681_MOESM1_ESM.pdf]

ENCUESTA SOBRE LA APLICACIÓN DE LA MEDICINA BASADA EN EVIDENCIAS EN ESTUDIANTES DE MEDICINA Y PERSONAL MÉDICO

*La información obtenida en el presente cuestionario es confidencial y será utilizada únicamente con fines de investigación.

Sexo: M ☐ F ☐ **Universidad de procedencia:** ____________________

a) Médico de Base: ☐

____
Especialidad

c) Médico Interno de Pregrado: ☐

b) Estudiante de Medicina: ☐

d) Médico Residente: ☐

____
Especialidad

Año que cursa ☐

1. ¿Está usted familiarizado con la Medicina Basada en la Evidencia?

Mucho ☐ Regular ☐ Un poco ☐ Nada ☐

2. ¿Cómo la aprendió?

En la escuela de medicina ☐ En cursos sobre este tema ☐ Estudiando por mi cuenta ☐

3. ¿Considera usted que esta disciplina es para el clínico?

Muy importante ☐ Regularmente importante ☐ No tiene importancia ☐

4. ¿Cómo definiría usted a la Medicina Basada en la Evidencia?

____

____

____

____

5. ¿Podría usted mencionar cuales son las fases o los pasos que deben tomarse en cuenta para hacer Medicina Basada en la Evidencia?

a. ____

b. ____

c. ____

d. ____

e. ____

6. ¿Usted considera que aplica la Medicina Basada en la Evidencia en su práctica médica habitual?:

Siempre ☐ Generalmente ☐ A veces ☐ Nunca ☐

7. ¿En qué forma aplica usted la Medicina Basada en la Evidencia?

____

____

____

____
